# COVID-19 and regional inequalities in childhood vaccination uptake in England: a spline regression

**DOI:** 10.1186/s12889-025-24207-9

**Published:** 2025-08-25

**Authors:** Amber Sacre, Sarah Sowden, Clare Bambra, Natalie Bennett, Adam Todd

**Affiliations:** 1 Population Health Sciences Institute, Newcastle University, Newcastle, NE1 7RU UK; 2 National Institute for Health and Care Research (NIHR) Applied Research Collaboration (ARC) North East and North Cumbria (NENC), Newcastle, NE3 3XT UK; 3 Department of Sociology, University of Manchester, Manchester, M13 9PL UK; 4 School of Pharmacy, Newcastle University, Newcastle, NE1 7RU UK; 5 Newcastle Patient Safety Research Collaboration (PSRC), Newcastle University, Newcastle, NE1 7RU UK

**Keywords:** COVID-19, Health inequalities, Socioeconomic inequalities, Vaccine uptake, Childhood vaccination, Spline regression

## Abstract

**Background:**

In recent years, the uptake of childhood vaccinations before the age of five has declined globally. In England, the decline in measles, mumps, and rubella (MMR) vaccination coverage is concerning and has subsequently been followed by an increase in measles outbreaks. This study analysed the sustained and additional impact of the COVID-19 pandemic on area-level inequalities in childhood vaccination uptake across regions in England.

**Methods:**

Spline regressions with three-way interaction terms were used to assess the impact of COVID-19, from the first lockdown, on local authorities across regions and deprivation quartiles. Cover of Vaccination Evaluated Rapidly data was used for the uptake statistics, and the Indices of Multiple Deprivation 2019 for the deprivation quartiles. The lowest coverage vaccines were analysed: the pre-school booster (one dose) and MMR (two doses, cumulative) from July – September 2014 to October – December 2022.

**Results:**

The findings suggest the odds of childhood vaccination uptake were declining in England prior to the first lockdown, but there was an additional decrease associated with this event by 12% (OR:0.88, CI:0.79–0.99, *p* < .05) for the pre-school booster and 13% (OR:0.87, CI:0.77–0.99, *p* < .05) for the MMR vaccine. For local authorities classified as the most deprived 25% (Quartile 1), there was a 15% (OR: 0.85, CI: (0.72–0.99, *p* < .05) post-lockdown-associated decline in the odds of MMR uptake, but not the pre-school booster. Post-lockdown and region interaction effects for the pre-school booster were as follows: Yorkshire and the Humber, 39% decreased odds (OR:0.61, CI:0.54–0.68, *p* < .001); East Midlands, 22% decreased odds (OR:0.77 OR, CI:0.62–0.95, *p* < .05); and West Midlands, 19% increased odds (OR:1.19, CI:1.00-1.40, *p* < .05). For the MMR vaccine, these were: Yorkshire and the Humber, 41% decreased odds (OR:0.59, CI:0.52–0.67, *p* < .001); North West, 30% increased odds (OR:1.3, CI:1.04–1.64, *p* < .05); West Midlands, 21% increased odds (OR:1.21, CI:1-1.46, *p* < .05); and South East, 19% decreased odds (OR:0.67, CI:0.98, *p* < .05). Evidence of three-way post-lockdown interaction effects for deprivation quartile and region was also identified for both vaccines.

**Conclusions:**

The results highlight the need for national vaccination uptake analyses to consider regional variation, as similarly deprived local authorities do not necessarily exhibit the same COVID-19-associated effects.

**Supplementary Information:**

The online version contains supplementary material available at 10.1186/s12889-025-24207-9.

## Background

In recent years, global childhood vaccination uptake (those administered before the age of five) has been declining [1]. Despite preventing an estimated 57 million child deaths globally between 2000 and 2022, in 2022, the proportion of children vaccinated against measles by their first birthday was 83%, the lowest since 2008 [1]. Potential explanations involve a complex interplay between, for example, demand and supply-side factors, vaccine hesitancy, and the accessibility of vaccination services [2]. The decline in MMR (measles, mumps, and rubella) vaccination uptake in England has been described as “alarming” by members of parliament (MPs) [3] and, as a consequence, the decline has led to an increase in measles outbreaks [4]. In 2024, there were 2,911 laboratory-confirmed measles cases in England, the highest number of cases recorded annually since 2012 [4]. This was initially driven by an outbreak in Birmingham but was soon overtaken by a large outbreak in London, with small clusters in other regions. Case counts followed a downward trajectory from mid-July, with small, localised outbreaks continuing in some regions [4]. Measles-containing vaccines require 94% coverage to achieve herd immunity, one of the highest levels among all the vaccines, due to the disease’s high reproducibility rate [5]. Several respiratory, neurological, gastrointestinal, ophthalmic, hematologic, cardiovascular, and dermatologic complications are associated with contracting measles, with the most severe being mortality [6].

Moreover, marginalised communities can be more susceptible to lower vaccination uptake than non-marginalised communities [2]. Marginalised communities are those who are disadvantaged by societal power dynamics [7], such as individuals experiencing socioeconomic disadvantage. Much literature on health and healthcare inequalities refers to a social gradient, whereby, on average, those in a more socioeconomically advantaged position have better health, more healthcare opportunities and improved healthcare outcomes than those in a less advantaged position [8–13]. For example, a study conducted in Liverpool, UK, exploring a measles outbreak in 2012–13, identified that deprived neighbourhoods had the highest proportion of disease-susceptible children due to under-immunisation [14]. These pockets of low uptake can, in turn, exacerbate socioeconomic inequalities. This is furthered by the fact that childhood vaccination uptake is not consistent across regions, and each region has a unique socioeconomic profile [15]. For instance, London exhibits the lowest coverage, where 90% has not been achieved for any childhood vaccine in recent years [16].

The COVID-19 pandemic contributed to worsening socioeconomic inequalities in health and healthcare [17]. This event also refocused public attention on the topic of vaccination and may have contributed to an overall increase in vaccine hesitancy [18–22]. In the context of the pandemic, research suggests that for some vaccines, uptake in England, although already declining, was additionally impacted by the introduction of physical distancing measures. However, this finding was not consistent across all childhood vaccines [23]; hypothesised as linked to the fluctuating nature of pandemic restrictions and regulations on accessing public settings, including healthcare, coupled with the varying timeframes in which each childhood vaccination needs to be administered [23]. Evidence of lower MMR vaccination uptake for disadvantaged groups compared to all other childhood vaccines was identified over the course of the pandemic [24]. Further research has shown an overall decline of 4% in MMR vaccination uptake in North East London, although children living in the most disadvantaged areas who did receive the vaccine were *more* likely to receive it on time compared to those in less disadvantaged areas [25]. Indeed, other research not related to the COVID-19 pandemic suggests that inverse associations may exist, where low childhood vaccination uptake is found in more advantaged socioeconomic groups [2, 26, 27].

In summary, vaccination uptake is multifaceted. In view of: [1] declining childhood vaccination uptake [2], increasing vaccine-preventable disease outbreaks [3], clear regional trends in coverage [4], worsening post-COVID-19 socioeconomic inequalities in health and healthcare, and [5] complex associations between socioeconomic factors and uptake, further research into the culmination of these facets is warranted. Existing quantitative studies do not adequately assess the sustained impact of COVID-19 on regional inequalities in childhood vaccination uptake [24]. A greater understanding of these associations has the potential to support policy, practice, and decision-making with regard to the implementation of childhood vaccination programmes. In order to maximise their reach and effectiveness, “health actions [interventions] must be universal, not targeted, but with a scale and intensity that is proportionate” [28, p. 1]]. This study, therefore, aimed to analyse whether the COVID-19 pandemic had sustained an additional impact on area-level inequalities in childhood vaccination uptake across regions in England.

## Methods

Spline regressions with three-way interaction terms were used to assess the impact of COVID-19, from the first national lockdown, on childhood vaccination uptake in local authorities across regions, and deprivation quartiles, in England, from July – September 2014 to October – December 2022. The model specification is presented in Supplementary Material S1. The ‘Cover of Vaccination Evaluated Rapidly’ (COVER) data were used for uptake statistics [16], and the Indices of Multiple Deprivation (IMD) from the English Indices of Deprivation (IoD) 2019 [29] were used for the deprivation quartiles. The vaccines analysed were the pre-school booster (one dose) and MMR (two doses, cumulative) [30].

### Data

COVER data are official statistical reports published quarterly and annually according to the financial year. Routine data is most useful for population studies because it is often the best representation of the phenomena under study [31]. Additionally, COVER undergoes several phases of verification [32], albeit with some persisting discrepancies evidenced by the caveat tables provided with each dataset. Upper-tier local Authority-level (UTLA) data were used, and uptake was operationalised as a binary variable: vaccinations administered (success) versus eligible children in a local authority (total trials). The uptake of the pre-school booster and MMR vaccines was analysed, as these vaccines consistently exhibit the lowest coverage of all childhood vaccines [32–34].

COVER data is not published in the quarter following the recording of uptake and, instead, reflects the quarters in which each cohort reached their first, second, and fifth birthday, known as the “evaluation quarter”, meaning a lag is required for real-time analysis. Statistical publications from 2016 to the present day were selected, as potential data quality issues existed prior to 2016, when data collection was still considered experimental [35]. Thus, there were 34 time points in total, from April – June 2016 to July – September 2024, which, as a lag is required, reflect vaccines administered 6 or 7 quarters prior, meaning the timeframe is July – September 2014 to October - December 2022 (see Supplementary Material S2 for more information). This provides 33 observation points, as the data for the April-June 2021 evaluation quarter (reflecting vaccination uptake in September-November 2019) has not been published. For the purpose of our analysis, these data were treated as Missing at Random (MAR); the absence of the data does not dictate the likelihood of the outcome [36].

The IMD was used to assess area-level deprivation, a product of the English IoD 2019 [29]. It provides measures of deprivation at lower-layer super-output areas (LSOA), which are collections of 400 to 1,200 households. The IMD is based on seven domains: income, employment, education, health and disability, crime, housing and services, and living environment. Thus, the index comprehensively describes the socioeconomic characteristics of an area. UTLA data were used to ensure the geographical granularity converged with the vaccination uptake statistics, specifically, the “average rank” measure. These were divided into four categories and represent the deprivation quartiles used in the analyses – this approach balances theoretical rationalisation (the CORE20PLUS5 NHS England strategy for reducing healthcare inequalities uses quintiles of deprivation, focusing on the 20% most deprived [37]) and statistical clarity. Operationalisations with more categories, such as quintiles or deciles, introduce rank deficiencies and, subsequently, a greater prevalence of unavailable data, underpinning our decision to use quartiles instead.

To ensure consistency of UTLAs across the analysed timeframe, some aggregations were required for North and West Northamptonshire, Hackney and the City of London, and Bournemouth and Poole. The data cleaning process meant 150 local authorities were analysed consistently across 34 time points, with one quarter missing (April-June 2021), resulting in 4,950 observations.

### Analyses

**Fig. 1:**
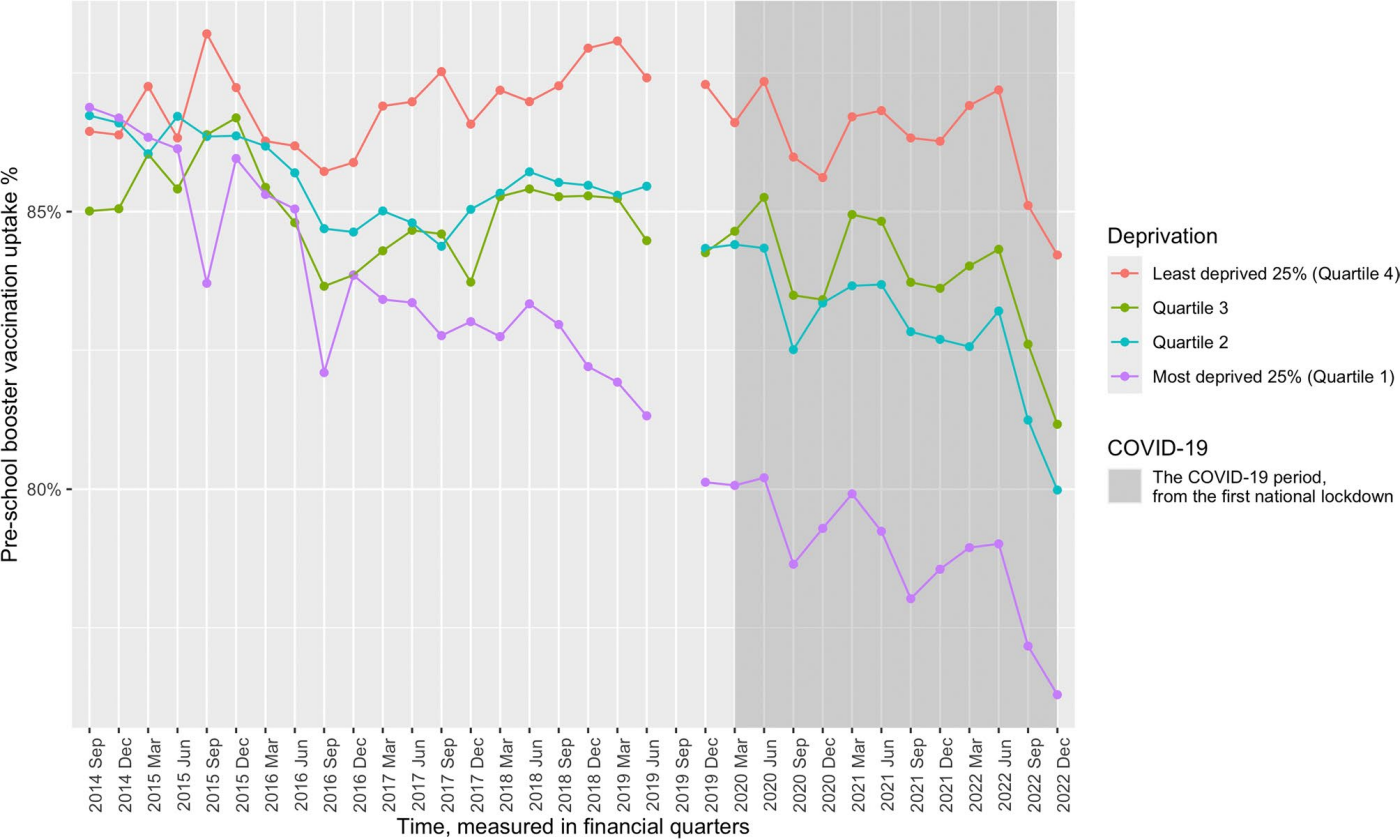
Line graph of pre-school booster vaccination coverage across deprivation quartiles from 2014–2022. September 2019 is missing data

**Fig. 2:**
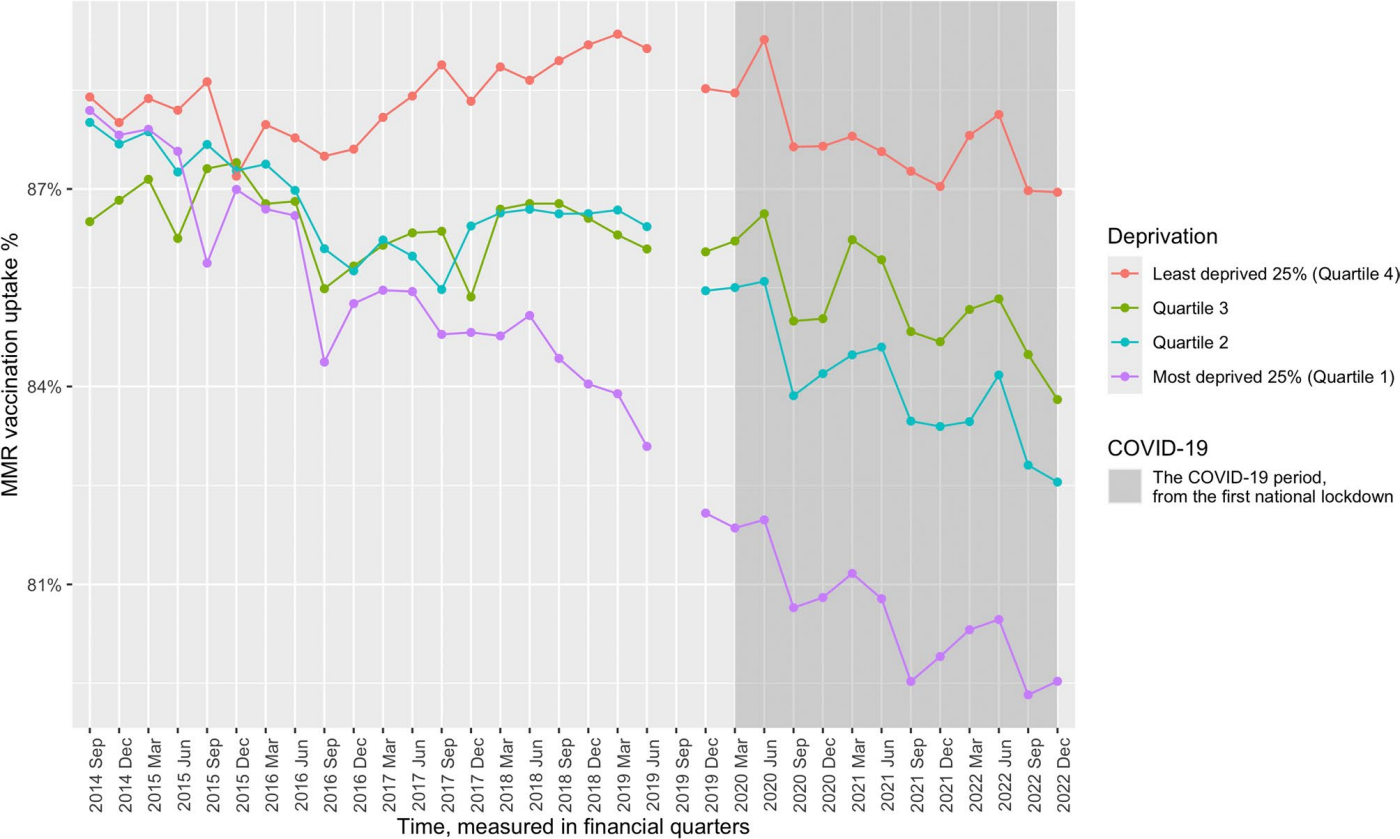
Line graph of MMR (measles, mumps, and rubella) vaccination coverage across deprivation quartiles from 2014–2022. September 2019 is missing data

Spline regressions, an increasingly common test for the impact of policies or interventions at a population level [38, 39], were employed for the analysis. The breakpoint investigated was the first lockdown, defined as a state of emergency where movement from the home was restricted [40]. Thus, access to childhood vaccination services was limited, and the topic of vaccination, particularly in reference to the COVID-19 vaccine, was gaining prominence in the public consciousness. Additionally, this was a national policy that took effect simultaneously across the entire country, providing a clearly defined breakpoint location for the analyses. As the first lockdown came into effect at the end of a financial quarter (January – March 2020), the following quarter (April – June 2020) was used as the breakpoint to account for the lag in impact. This was operationalised as a cubic spline term to account for the evident non-linearity in childhood vaccination uptake when analysing time trends (refer to Figs. 1 and 2).

UTLAs were grouped by region to enable comparisons across England (London, South East, South West, East of England, East Midlands, West Midlands, North East, Yorkshire and the Humber, and North West). Due to the lack of UTLAs classified as each of the deprivation quartiles for all regions, estimates could not be calculated for some quartile and region combinations.

Robust standard errors clustered by UTLA were employed to control for the non-independence of observations, ensuring a more reliable interpretation of the coefficients. Model comparisons and further tests were performed to assess the robustness of the models and the appropriateness of our methodological decisions, such as:

- Interchanging fixed effects for random effects (Supplementary Material S3).
- Using continuous operationalisations of deprivation (Supplementary Material S4).
- Exchanging reference categories (Supplementary Material S5 and S6).
- Exchanging cubic spline terms for linear spline terms (Supplementary Material S7).

RStudio (v. 2024.12.1) was used to perform the analyses [41].

## Results

### Descriptive analysis results

The England average uptake for the pre-school booster across the study period (July–September 2014 to October–December 2022) was 86.05% (Min = 82.72% in December 2021, max = 87.94% in March 2015). MMR vaccine uptake followed a similar trend to the pre-school booster, averaging 87.39% (Min = 84.93% in September 2022, Max = 89.05% in March 2015).

Figure 1 illustrates the trends in pre-school booster coverage across the study period. Among local authorities classified as the most deprived 25% (quartile 1), there is an evident decline in coverage, with highs of 86.9% and lows of 76.3%, demonstrating an absolute decrease of 10.6%. While quartiles 2, 3 and 4 also show a decline in coverage, there is less variation: quartile 2 = 80-86.7% (6.7% absolute decline), quartile 3 = 81.2–86.7% (5.5% absolute decline), and quartile 4 = 84.2–88.2% (4.0% absolute decline).

Figure 2 illustrates the trends in MMR vaccination coverage across the study period. The most deprived 25% (quartile 1) local authorities had highs of 88.2% and lows of 79.3%, indicating an absolute decline of 8.9%. Quartiles 2, 3, and 4 local authorities demonstrated similar declines in coverage: quartile 2 = 82.6–88% (5.5% absolute decline), quartile 3 = 83.8–87.4% (3.6% absolute decline), quartile 4 = 87-89.4% (2.4% absolute decline).

Table 1 presents pre-school booster and MMR vaccination coverage averages across IMD deprivation quartiles and regions. The North East of England was the only region that achieved an average of 90% pre-school booster coverage across the study period. Seven regions averaged above 85%, aside from London, with a mean of 70.9%. Regarding the MMR vaccine, the North East and South West averaged above 90% coverage; six of the remaining seven regions of England averaged above 87%, aside from London, which averaged 74.9% coverage.

**Table 1:** Pre-school booster and MMR vaccination coverage averages according to deprivation quartile and region

Pre-school booster	Least deprived 25%	Quartile 3	Quartile 2	Most deprived 25%	Mean	Min-Max
	Quartile 4			Quartile 1		
North East	NA	90.7%	90.2%	89.2%	90.1%	86.0–93.6
Yorkshire & Humber	89.5%	87.2%	89.1%	88.5%	88.2%	81.0–92.2
North West	90.9%	89.6%	87.9%	85.2%	88.3%	79.8–94.8
East Midlands	91.8%	87.8%	81.9%	80.0%	85.1%	71.4–96.1
West Midlands	88.9%	89.6%	87.2%	83.3%	87.5%	78.1–94.6
South West	91.4%	88.6%	88.3%	89.3%	89.1%	84.3–93.7
South East	88.1%	84.1%	85.0%	NA	85.7%	80.4–91.1
East of England	88.7%	88.1%	80.3%	NA	85.9%	75.1–91.0
London	75.8%	69.5%	72.7%	71.6%	70.9%	58.2–81.0
**Mean**	88.1%	86.1%	84.7%	83.9%		
**Min-Max**	75.8–91.8	69.5–90.7	72.7–90.2	71.6–89.3		

MMR vaccine	Least deprived 25%	Quartile 3	Quartile 2	Most deprived 25%	Mean	Min-Max
	Quartile 4			Quartile 1		
North East	NA	92.1%	91.3%	90.2%	90.9%	87.9–94.0
Yorkshire & Humber	90.2%	88.0%	90.2%	88.8%	88.9%	82.0–92.4
North West	91.8%	90.6%	88.8%	86.6%	89.5%	81.8–95.5
East Midlands	93.0%	88.5%	83.0%	82.2%	86.5%	74.5–97.6
West Midlands	89.7%	90.3%	88.3%	84.7%	88.5%	79.4–94.3
South West	92.8%	90.5%	89.4%	91.6%	90.8%	86.8–95.6
South East	89.5%	86.7%	86.5%	NA	87.7%	83.9–91.9
East of England	90.0%	89.0%	82.6%	NA	87.2%	77.1–91.6
London	78.2%	71.6%	73.7%	74.5%	74.2%	64.9–83.2
**Mean**	89.4%	87.5%	86.0%	85.5%		
**Min-Max**	78.2–93	71.6–92.1	73.7–91.3	74.5–91.6		

*NA* = data not available; no local authorities in region classified as deprivation quartile

### Spline regression results: two-way COVID-19-associated interaction effects

As shown in the descriptive analysis, both pre-school booster and MMR vaccination coverage were already declining prior to COVID-19 and the first national lockdown. The spline regression captures how the odds of vaccination uptake and regional inequalities shifted during the post-lockdown period, beyond the pre-existing downward trend.

The following results are outlined in Table 2. There was an overall 12% (OR: 0.88, CI: 0.79–0.99, *p* >.05) additional, sustained decrease in the odds of pre-school vaccination uptake post-lockdown. No significant two-way interaction effects were identified between post-lockdown and deprivation quartile. However, significant two-way interaction effects for post-lockdown and region, when compared to London (reference category), were exhibited by: Yorkshire and the Humber, 39% decreased odds of pre-school booster uptake (OR: 0.61, CI: 0.54–0.68, *p* <.001); East Midlands, 23% decreased odds (OR: 0.77 OR, CI: 0.62–0.95, *p* <.05); and West Midlands, 19% increased odds (OR: 1.19, CI: 1.00-1.4, *p* <.05).

For the MMR vaccine, a similar overall post-lockdown-associated 13% (OR: 0.87, CI: 0.77–0.99, *p* <.05) decrease in the odds of uptake was observed. Two-way interaction effects were identified between post-lockdown and deprivation quartile, with the most disadvantaged 25% (Quartile 1) of local authorities experiencing a 15% (OR: 0.85, CI: 0.72–0.99, *p* <.05) decreased odds of MMR vaccination uptake associated with this event. Significant additional post-lockdown-associated changes in MMR vaccination uptake were found for: Yorkshire and the Humber, 41% decreased odds (OR: 0.59, CI: 0.52–0.67 *p* <.001); North West, 30% increased odds (OR: 1.3, CI: 1.04–1.64, *p* <.05); West Midlands, 21% increased odds (OR: 1.21, CI: 1-1.46, *p* <.05); and South East, 19% decreased odds (OR: 0.81, CI: 0.67–0.98, *p* <.05).

**Table 2:** Spline regression results. Two-way post-lockdown-associated changes in pre-school booster and MMR vaccination uptake from 2014–2022 according to deprivation quartile and regional location

Two-way post-lockdown-associated interaction effects^a^						
	Pre-school Booster			MMR		
(ref. London, Quartile 3)	*OR (95% CI)*			*OR (95% CI)*		
Pre-lockdown	0.66	(0.34–1.30)		0.63	(0.31, 1.27)	
Post-lockdown	0.88	(0.79–0.99)	*	0.87	(0.77–0.99)	*
Local authorities classified as the…						
…least deprived 25% (Quartile 4)	0.92	(0.76–1.11)		0.94	(0.78, 1.14)	
…Quartile 2	0.94	(0.75–1.17)		0.97	(0.75–1.26)	
…most deprived 25% (Quartile 1)	0.92	(0.78–1.09)		0.85	(0.72–0.99)	*
Local authorities in the…						
…North East	1.06	(0.69–1.64)		1.04	(0.67–1.62)	
…Yorkshire and the Humber	0.61	(0.54–0.68)	***	0.59	(0.52–0.67)	***
…North West	1.07	(0.92–1.25)		1.3	(1.04–1.64)	*
…East Midlands	0.77	(0.62–0.95)	*	0.81	(0.65–1.01)	
…West Midlands	1.19	(1.00-1.40)	*	1.21	(1-1.46)	*
…South West	0.94	(0.73–1.21)		0.86	(0.7–1.06)	
…South East	0.93	(0.75–1.14)		0.81	(0.67–0.98)	*
…East of England	0.88	(0.70–1.12)		0.9	(0.7–1.15)	

^a^The estimates reported in this table are the result of two-way interactions:(1) post-lockdown and deprivation quartile; (2) post-lockdown and region

* *p* ≤.05, ** *p* ≤.01, *** *p* ≤.001

### Spline regression results: three-way COVID-19-associated interaction effects

The following results are reported in Table 3, and in graphical form in Supplementary Materials S8 and S9. Three-way interaction effects between post-lockdown, deprivation quartile and region exhibited several significant associations for pre-school booster uptake. For local authorities classified as the least deprived 25% (Quartile 4), those in Yorkshire and the Humber experienced a 96% (OR: 1.96, CI: 1.45–2.66, *p* <.001) increased odds of pre-school booster uptake, compared to local authorities in Quartile 3, London, whereas the West Midlands observed a 22% (OR: 0.78, CI: 0.61–0.99, *p* <.05) decrease. Amongst local authorities classified as Quartile 2 in the North West, there was a 32% (OR: 0.68, CI: 0.49–0.96, *p* <.05) decreased odds of pre-school booster uptake, compared to local authorities in Quartile 3, London, similar to the West Midlands, which experienced a 27% (OR: 0.73, CI: 0.54–0.98, *p* <.05) decreased odds. Lastly, evidence suggests local authorities classified as Quartile 1 in the North West and West Midlands observed decreased odds of pre-school booster uptake by 42% (OR: 0.58, CI: 0.45–0.75, *p* <.001) and 33% (OR: 0.67, CI: 0.54–0.82, *p* <.001), respectively, compared to local authorities in Quartile 3, London.

Regarding the MMR vaccine, amongst local authorities classified as Quartile 4, there was evidence of post-lockdown-associated effects in some regions compared to Quartile 3, London: Yorkshire and the Humber, a 84% (OR: 1.84, CI: 1.35–2.51, *p* <.001) increased odds of MMR vaccination uptake; West Midlands, a 25% (OR: 0.75, CI: 0.59–0.96, *p* <.05) decreased odds of MMR vaccination uptake; South East, a 45% (OR: 1.04, CI: 1.04–2.04, *p* <.05) increased odds of MMR vaccination uptake. Moreover, there was evidence of post-lockdown-associated decreased odds of MMR vaccination uptake for local authorities classified as Quartile 2 (when compared to London and Quartile 3) for the North West (37% decreased odds (OR: 0.63, CI: 0.43–0.92, *p* <.05)). Similarly, for local authorities classified as Quartile 1, post-lockdown-associated effects were identified for the North West and West Midlands, with 46% (OR: 0.54, CI: 0.41–0.71, *p* <.001) and 28% (OR: 0.72, CI: 0.58–0.89, *p* <.01) decreased odds of MMR vaccination uptake, respectively, compared to Quartile 3, London.

**Table 3:** Spline regression results. Three-way post-lockdown-associated changes in pre-school booster and MMR vaccination uptake from 2014–2022 according to deprivation quartile and regional location

Three-way post-lockdown-associated interaction effects^a^						
(ref. London, Quartile 3)	Pre-school Booster			MMR Vaccine		
	*OR (95% CI)*			*OR (95% CI)*		
Quartile 4 local authorities in the…						
…North East	NA^2^			NA^2^		
…Yorkshire and the Humber	1.96	(1.45–2.66)	***	1.84	(1.35–2.51)	***
…North West	0.82	(0.51–1.30)		0.77	(0.51–1.17)	
…East Midlands	0.88	(0.67–1.16)		0.82	(0.63–1.07)	
…West Midlands	0.78	(0.61–0.99)	*	0.75	(0.59–0.96)	*
…South West	1.13	(0.82–1.55)		1.23	(0.94–1.61)	
…South East	1.26	(0.91–1.75)		1.45	(1.04–2.04)	*
…East of England	1.08	(0.80–1.44)		1.07	(0.79–1.44)	
Quartile 2 local authorities in…						
…North East	0.73	(0.45–1.18)		0.68	(0.41–1.12)	
…Yorkshire and the Humber	1.18	(0.91–1.53)		1.21	(0.88–1.65)	
…North West	0.68	(0.49–0.96)	*	0.63	(0.43–0.92)	*
…East Midlands	1.27	(0.95–1.68)		1.23	(0.90–1.68)	
…West Midlands	0.73	(0.54–0.98)	*	0.74	(0.51–1.07)	
…South West	0.95	(0.69–1.30)		0.98	(0.71–1.37)	
…South East	0.98	(0.67–1.45)		0.97	(0.68–1.37)	
…East of England	1	(0.67–1.49)		0.83	(0.56–1.22)	
Quartile 1 local authorities in…						
…North East	0.86	(0.53–1.37)		0.94	(0.56–1.57)	
…Yorkshire and the Humber	0.98	(0.69–1.41)		1.16	(0.82–1.63)	
…North West	0.58	(0.45–0.75)	***	0.54	(0.41–0.71)	***
…East Midlands	0.84	(0.65–1.09)		0.87	(0.67–1.13)	
…West Midlands	0.67	(0.54–0.82)	***	0.72	(0.58–0.89)	**
…South West	0.88	(0.66–1.16)		0.9	(0.72–1.13)	
…South East	NA^2^			NA^2^		
…East of England	NA^2^			NA^2^		

NA^2^ = data not available; no local authorities in region classified as deprivation quartile

^a^The estimates reported in this table are the result of a three-way interaction: Post-lockdown, deprivation quartile, and region

* *p* ≤.05, ** *p* ≤.01, *** *p* ≤.001

## Discussion

This study used a spline regression approach to analyse whether the COVID-19 pandemic had an additional and sustained impact on area-level inequalities in childhood vaccination uptake across regions in England. There are three main findings of this study. Firstly, there was an overall decline in the odds of pre-school booster and MMR vaccination uptake from July-September 2014 to October-December 2022, with an increased rate of decline associated with the post-lockdown period. Secondly, the descriptive statistics portray an overall widening of socioeconomic inequalities in pre-school booster and MMR vaccination uptake, supported by an overall post-lockdown-associated decline in the odds of MMR vaccination uptake identified in the spline regression for the most deprived 25% of local authorities, but this was not found for the pre-school booster vaccination. Lastly, similarly classified deprived local authorities across regions in England experienced post-lockdown-associated changes in uptake differently; Yorkshire and the Humber, the North West, East Midlands, West Midlands, and South East regions appeared most susceptible to these. While previous studies explore these topics individually – the decline in childhood vaccination uptake, including the additional effect of the COVID-19 pandemic [23–25, 42, 43]; the association between socioeconomic position and childhood vaccination uptake [2, 44]; and the disproportionately negative parental perceptions of the MMR vaccine [27, 45–48] – they lack an in-depth account of COVID-19, area-level inequalities, and childhood vaccination uptake in combination.

Adverse attitudes towards the MMR vaccine may stem from the now-retracted article published in 1998 by Andrew Wakefield [49], linking uptake to the onset of autism spectrum disorder. The effect of the Wakefield article was far-reaching; there is evidence to suggest an increase in vaccine hesitancy, a decrease in vaccine uptake, and a subsequent increase in Measles outbreaks [47, 50, 51]. However, the impact expanded beyond Measles, with “spillover” effects contributing to a decrease in the uptake of other childhood vaccinations [27]. Potential “spillover” effects may be at play when considering the impact of COVID-19 on childhood vaccination. Commissioners and providers of vaccinations must be aware of the possible impact (un)favourable perceptions of vaccines may have on the uptake of, and attitudes towards, other vaccinations in the routine schedule. In the event of new vaccines being introduced to the childhood vaccination schedule, action may need to be taken to safeguard current uptake levels, given people may have less confidence in a new vaccine – especially if it uses new technology (e.g. an mRNA vaccine).

The findings also suggest the association between COVID-19, childhood vaccination uptake and area-level inequalities across English regions is complex. The theory of “deprivation amplification” posits that individuals of a disadvantaged socioeconomic position experience (relative) “amplified” effects when living in areas with a prevalence of deprivation [52, 53]. In their report, *Health Equity in England: The Marmot Review 10 Years On*, the authors identified stark regional differences in life expectancy for individuals living in the most disadvantaged deciles in more overall deprived regions [54]. However, the findings from this study do not necessarily adhere to this pattern. Indeed, the North East of England had the highest overall uptake of childhood vaccinations compared to any other English region; the North East has been specifically cited in the previous report as being one of the most disadvantaged regions in England. The phenomenon where areas with greater levels of socioeconomic advantage but lower rates of vaccination is known as the “privilege paradox” and has been discussed in the wider literature in an Australian context [55]. The authors suggested that uptake was heavily influenced by geographical location. Similar findings were demonstrated in our study, supporting the notion that a “privilege paradox” can exist in vaccination uptake at the area-level. However, in the context of England, this is contradictory to common debates regarding socioeconomic gradients in health and healthcare [9, 56] and North-South health divides [57–60]. This suggests there may be other causal mechanisms which interact with area-level inequalities and vaccination uptake. For example, high levels of trust in vaccines and vaccination providers are cited as promoters of uptake [2, 61–63], but trust in healthcare providers is multifactorial and varies across England [64]. Other explanations may be linked to the demographic composition of a region and the prevalence of those who may face more barriers to healthcare access, such as ethnic minority groups [65]. These may manifest differently across regions.

The findings of this study have various applications. Public health and healthcare policymakers in England can use these insights on regional patterns of vaccination uptake before and after the COVID-19 pandemic to inform more equitable resource allocation. This is particularly important for areas with lower rates of childhood vaccination and a subsequent greater risk of vaccine-preventable disease outbreaks. Also, regional-level public health and healthcare policymakers can consider how they perform compared to other regions. This comparison may foster communication and promote learning on effective strategies to improve vaccination uptake, especially in socioeconomically disadvantaged populations. Indeed, research suggests successful interventions are complex and targeted towards the barriers and needs of groups with low uptake [66]. More broadly, the findings of this study can encourage other countries to explore vaccination uptake at various levels of geographic granularity to gain a more intimate understanding of where uptake is high or low, and why.

### Limitations

This study has three main limitations. Firstly, using area-level data in place of individual-level data can introduce issues of ecological fallacy when interpreting the results [67]. Area-level analysis does not reflect the nuance that exists at the individual level, as it can cause extreme inequality to appear smoother, and can inflate the risk of omitted variable bias [67]. Thus, our analysis does not capture these extremes like a more granular approach would. However, one study found that individual-level data did not identify associations between sociodemographic factors and human papillomavirus vaccination uptake that were present at the area level [68]. This demonstrates the usefulness of analysing uptake data at the area level, as it can aid in understanding why trends are seen in specific locations. Secondly, this study used quarterly uptake data, causing some convergence issues. There are discrepancies between the publication (or “evaluation”) quarter of COVER data and the quarter in which vaccination uptake occurred; the evaluation quarter reflects vaccination administered 20 months prior. Thus, the data needed to be lagged by six or seven quarters. A lag of seven quarters was chosen for the analyses because this would correctly converge with two-thirds of eligible children. Lastly, two potentially relevant considerations were absent from the analysis and discussion. It is acknowledged that uptake was already declining before the onset of COVID-19; however, this study did not examine those earlier trends. Also, we did not quantify how much socioeconomic inequalities in vaccination uptake widened. Exploring these pre-pandemic declines and quantifying the inequalities in coverage could provide valuable insight into the factors contributing to the downward trend.

### Suggestions for future research

Future research could statistically explore the impact of other socio-political events prior to the COVID-19 pandemic to understand what may have initiated and contributed to the pre-existing decline in childhood vaccination uptake. Additionally, an exploration of the causal mechanisms linking area-level inequalities and vaccination uptake is warranted. This could have a specific focus on the North East of England, to try to understand the reasons why vaccination uptake was so high compared to the other English regions. The theory of deprivation amplification appears to hold for other regions with a high prevalence of area-level inequalities, such as Yorkshire and the Humber and the North West, aside from the North East (in the context of childhood vaccination). Probing into potential reasons for this may be beneficial in understanding whether the region has valuable learning that can be applied elsewhere. In summary, our study highlights the need for national vaccination uptake research to consider regional variation.

## Conclusions

In conclusion, uptake of both the pre-school booster and MMR vaccine declined over the study period (July-September 2024 to October-December 2022) even before the onset of COVID-19. Evidence of widening socioeconomic inequalities in MMR vaccination coverage, exacerbated by the pandemic, was identified. The findings also revealed that regions across England experienced changes in childhood vaccination uptake post-lockdown in different ways, and this event did contribute to an increased rate of decline in some areas. Furthermore, the results highlight the need for national vaccination uptake analyses to consider regional variation, as similarly deprived local authorities do not necessarily exhibit the same post-lockdown-associated effects. This emphasises the importance of recognising and addressing local and regional health inequalities in the context of childhood vaccination uptake in England.

## Supplementary Information

Supplementary Material 1.

## Data Availability

The datasets generated and/or analysed during the current study are available in the UK Health Security Agency repository, https://www.gov.uk/government/collections/vaccine-uptake.

## Abbreviations

UKHSA: UK Health Security Agency
MMR: Measles, mumps, and rubella vaccine
MPs: Members of Parliament
COVER: Cover of Evaluated Rapidly
IMD: Indices of Multiple Deprivation
UTLA: Upper-tier Local Authority
IoD: English Indices of Deprivation
JCVI: Joint Committee on Vaccination and Immunisation
