# Supplementary material for: COVID-19 and regional inequalities in childhood vaccination uptake in England: a spline regression
Source: BMC Public Health. 2025 Aug 25;25:2913. doi: 10.1186/s12889-025-24207-9 (PMC12376395; doi:10.1186/s12889-025-24207-9)

**Supplementary Material**

S1: Model specification.

${logit(Uptake}_{LA})=a_{0}+\beta_{1}LockdownSpline+$

$$\sum_{i=2}^{9} \beta_{2i}LockdownSpline*{Region}_{i}+$$

$$\sum_{j\in\left\{ 1,2,4,5 \right\}} \beta_{3i}LockdownSpline*{DepQuartile}_{j}+$$

$\sum_{i=2}^{9} \sum_{j\in\{1,2,4,5\}} \beta_{2i}LockdownSpline*{Region}_{i}*{DepQuartile}_{j}+$

$u_{LA}+ \varepsilon$

Where ${logit(Uptake}_{LA})$ is the number of children vaccinated in a local authority $LA$ offset by the total number of eligible children in $LA$. $a_{0}$ is the constant term, and$LockdownSpline$ is a third degree polynomial spline term with a breakpoint, or “knot”, when *time* (financial quarters) = 22, indicating the first national COVID-19 lockdown in England. ${DepQuartile}_{j}$ indicates the deprivation quartile (${DepQuartile}_{Ref}=3=$ “Quartile 3”) of $LA$ according to the 2019 Indices of Multiple Deprivation “average rank” metric. ${Region}_{i}$ is the regional indicator (${Region}_{Ref}=1=$ “London”). $u_{LA}$ and $\varepsilon$ are both error terms. $u_{LA}$ is the local authority time invariant local error term, and $\varepsilon$ is the idiosyncratic random error term. The model includes main effects of the $LockdownSpline$ but not for $Region$ or $DepQuartile$, because they are time invariant and their effects are absorbed by the panel structure.

S2: Data cleaning details.

| **Evaluation quarters** | | **Time Points** | **Uptake Quarters** | |
| --- | --- | --- | --- | --- |
| 2016-2017 | April-June | 0 | 2014-2015 | July-September |
| 2016-2017 | July-September | 1 | 2014-2015 | October-December |
| 2016-2017 | October-December | 2 | 2014-2015 | January-March |
| 2016-2017 | January-March | 3 | 2015-2016 | April-June |
| 2017-2018 | April-June | 4 | 2015-2016 | July-September |
| 2017-2018 | July-September | 5 | 2015-2016 | October-December |
| 2017-2018 | October-December | 6 | 2015-2016 | January-March |
| 2017-2018 | January-March | 7 | 2016-2017 | April-June |
| 2018-2019 | April-June | 8 | 2016-2017 | July-September |
| 2018-2019 | July-September | 9 | 2016-2017 | October-December |
| 2018-2019 | October-December | 10 | 2016-2017 | January-March |
| 2018-2019 | January-March | 11 | 2017-2018 | April-June |
| 2019-2020 | April-June | 12 | 2017-2018 | July-September |
| 2019-2020 | July-September | 13 | 2017-2018 | October-December |
| 2019-2020 | October-December | 14 | 2017-2018 | January-March |
| 2019-2020 | January-March | 15 | 2018-2019 | April-June |
| 2020-2021 | April-June | 16 | 2018-2019 | July-September |
| 2020-2021 | July-September | 17 | 2018-2019 | October-December |
| 2020-2021 | October-December | 18 | 2018-2019 | January-March |
| 2020-2021 | January-March | 19 | 2019-2020 | April-June |
| 2021-2022 | April-June | 20 | 2019-2020 | July-September |
| 2021-2022 | July-September | 21 | 2019-2020 | October-December |
| 2021-2022 | October-December | 22 | 2019-2020 | January-March^1^ |
| 2021-2022 | January-March | 23 | 2020-2021 | April-June^2^ |
| 2022-2023 | April-June | 24 | 2020-2021 | July-September |
| 2022-2023 | July-September | 25 | 2020-2021 | October-December |
| 2022-2023 | October-December | 26 | 2020-2021 | January-March |
| 2022-2023 | January-March | 27 | 2021-2022 | April-June |
| 2023-2024 | April-June | 28 | 2021-2022 | July-September |
| 2023-2024 | July-September | 29 | 2021-2022 | October-December |
| 2023-2024 | October-December | 30 | 2021-2022 | January-March |
| 2023-2024 | January-March | 31 | 2022-2023 | April-June |
| 2024-2025 | April-June | 32 | 2022-2023 | July-September |
| 2024-2025 | July-September | 33 | 2022-2023 | October-December |
| ^1^ First lockdown came into effect on 23^rd^ March 2020  ^2^ Breakpoint used in the analysis to represent the effects of the first national lockdown | | | | |

S3: Random effects.

|  | | | | | | |
| --- | --- | --- | --- | --- | --- | --- |
|  | **Pre-school Booster** | | | **MMR** | | |
| (ref. London, Quartile 3) | *OR (95% CI)* | | | *OR (95% CI)* | |  |
| **Pre-lockdown** | 0.87 | (0.84, 0.9) | *** | 0.87 | (0.83, 0.9) | *** |
| **Post-lockdown** | 0.96 | (0.94, 0.99) | ** | 0.97 | (0.94, 0.99) | ** |
| **Local authorities classified as the…** |  |  |  |  |  |  |
| …least deprived 25% (Quartile 4) | 0.99 | (0.95, 1.02) |  | 0.99 | (0.96, 1.03) |  |
| …Quartile 2 | 0.98 | (0.95, 1.02) |  | 0.99 | (0.96, 1.03) |  |
| …most deprived 25% (Quartile 1) | 0.98 | (0.95, 1.01) |  | 0.96 | (0.93, 0.99) | ** |
| **Local authorities in the…** |  |  |  |  |  |  |
| …North East | 1.03 | (0.98, 1.08) |  | 1.03 | (0.98, 1.08) |  |
| …Yorkshire and the Humber | 0.95 | (0.87, 1.03) |  | 0.95 | (0.87, 1.03) |  |
| …North West | 1.03 | (1.00, 1.06) |  | 1.05 | (1.01, 1.08) | ** |
| …East Midlands | 0.98 | (0.95, 1.02) |  | 0.99 | (0.96, 1.02) |  |
| …West Midlands | 1.04 | (1.00, 1.09) |  | 1.04 | (1.00, 1.08) |  |
| …South West | 1.01 | (0.98, 1.05) |  | 1.01 | (0.97, 1.04) |  |
| …South East | 1.01 | (0.97, 1.04) |  | 0.99 | (0.96, 1.02) |  |
| …East of England | 1.01 | (0.97, 1.04) |  | 1.01 | (0.97, 1.04) |  |
| **Quartile 4 local authorities in the…** |  |  |  |  |  |  |
| …North East | NA^2^ |  |  | NA^2^ |  |  |
| …Yorkshire and the Humber | 1.11 | (1.00, 1.22) | * | 1.09 | (0.99, 1.20) |  |
| …North West | 0.99 | (0.93, 1.05) |  | 0.98 | (0.92, 1.04) |  |
| …East Midlands | 1.02 | (0.96, 1.07) |  | 1.01 | (0.95, 1.06) |  |
| …West Midlands | 0.98 | (0.92, 1.03) |  | 0.97 | (0.92, 1.03) |  |
| …South West | 1.02 | (0.97, 1.07) |  | 1.02 | (0.98, 1.07) |  |
| …South East | 1.04 | (1.00, 1.08) |  | 1.05 | (1.01, 1.10) | * |
| …East of England | 1.01 | (0.97, 1.06) |  | 1.01 | (0.96, 1.05) |  |
| **Quartile 2 local authorities in…** |  |  |  |  |  |  |
| …North East | 0.98 | (0.92, 1.04) |  | 0.97 | (0.91, 1.03) |  |
| …Yorkshire and the Humber | 1.04 | (0.95, 1.14) |  | 1.04 | (0.95, 1.14) |  |
| …North West | 0.96 | (0.91, 1.01) |  | 0.95 | (0.91, 1.00) |  |
| …East Midlands | 1.03 | (0.96, 1.10) |  | 1.02 | (0.95, 1.10) |  |
| …West Midlands | 0.96 | (0.91, 1.02) |  | 0.97 | (0.91, 1.03) |  |
| …South West | 1.00 | (0.95, 1.05) |  | 1.00 | (0.95, 1.05) |  |
| …South East | 1.00 | (0.96, 1.06) |  | 1.00 | (0.95, 1.05) |  |
| …East of England | 0.98 | (0.92, 1.04) |  | 0.95 | (0.89, 1.01) |  |
| **Quartile 1 local authorities in…** |  |  |  |  |  |  |
| …North East | 1.00 | (0.94, 1.07) |  | 1.02 | (0.96, 1.09) |  |
| …Yorkshire and the Humber | 1.02 | (0.93, 1.12) |  | 1.05 | (0.95, 1.15) |  |
| …North West | 0.91 | (0.87, 0.95) | *** | 0.93 | (0.89, 0.97) | *** |
| …East Midlands | 0.94 | (0.89, 0.99) | * | 0.96 | (0.91, 1.02) |  |
| …West Midlands | 0.94 | (0.89, 0.98) | * | 0.96 | (0.91, 1.01) |  |
| …South West | 1 | (0.9, 1.11) |  | 1.02 | (0.92, 1.13) |  |
| …South East | NA^2^ |  |  | NA^2^ |  |  |
| …East of England | NA^2^ |  |  | NA^2^ |  |  |
| * *p* ≤ 0.05, ** *p* ≤ 0.01, *** *p* ≤ 0.001 | | | | | | |
| NA^2^ = data not available; no local authorities in region classified as deprivation quartile. | | | | | | |

S4: Continuous deprivation operationalisation.

|  | | | | | | |
| --- | --- | --- | --- | --- | --- | --- |
|  | **Pre-school Booster** | | | **MMR** | | |
| (ref. London, Quartile 3) | *OR (95% CI)* | | | *OR (95% CI)* | |  |
| **Pre-lockdown** | 0.34 | (0.22, 0.53) | *** | 0.36 | (0.25, 0.51) | *** |
| **Post-lockdown** | 0.78 | (0.69, 0.89) | *** | 0.74 | (0.65, 0.83) | *** |
| **IMD Rank** | 1.00 | (1.00, 1.00) |  | 1.00 | (1.00, 1.00) |  |
| **Local authorities in the…** |  |  |  |  |  |  |
| …North East | 0.80 | (0.57, 1.12) |  | 0.80 | (0.55, 1.17) |  |
| …Yorkshire and the Humber | 0.53 | (0.40, 0.71) | *** | 0.60 | (0.46, 0.78) | *** |
| …North West | 0.61 | (0.48, 0.78) | *** | 0.66 | (0.55, 0.80) | *** |
| …East Midlands | 0.72 | (0.57, 0.91) | ** | 0.77 | (0.59, 0.99) | * |
| …West Midlands | 0.81 | (0.71, 0.93) | ** | 0.86 | (0.76, 0.98) | * |
| …South West | 0.78 | (0.66, 0.93) | ** | 0.79 | (0.60, 1.04) |  |
| …South East | 0.76 | (0.51, 1.14) |  | 0.59 | (0.41, 0.86) | ** |
| …East of England | 0.90 | (0.64, 1.25) |  | 0.79 | (0.60, 1.04) |  |
| **IMD rank interaction effects in…** |  |  |  |  |  |  |
| …North East | 1.01 | (1.00, 1.01) |  | 1.00 | (0.99, 1.01) |  |
| …Yorkshire and the Humber | 1.00 | (1.00, 1.01) |  | 1.00 | (1.00, 1.01) | * |
| …North West | 1.00 | (1.00, 1.00) |  | 1.01 | (1.00, 1.01) | ** |
| …East Midlands | 1.00 | (1.00, 1.00) |  | 1.00 | (1.00, 1.00) |  |
| …West Midlands | 1.00 | (1.00, 1.00) |  | 1.00 | (1.00, 1.00) |  |
| …South West | 1.00 | (1.00, 1.01) |  | 1.00 | (1.00, 1.00) |  |
| …South East | 1.00 | (1.00, 1.00) |  | 1.00 | (1.00, 1.01) | * |
| …East of England | 1.00 | (0.99, 1.01) |  | 1.00 | (1.00, 1.00) |  |
| * *p* ≤ 0.05, ** *p* ≤ 0.01, *** *p* ≤ 0.001 | | | | | | |
| NA^2^ = data not available; no local authorities in region classified as deprivation quartile. | | | | | | |

S5: Exchanging deprivation reference categories.

|  | | | | | | |
| --- | --- | --- | --- | --- | --- | --- |
|  | **Pre-school Booster** | | | **MMR** | | |
| (ref. London, Quartile 1) | *OR (95% CI)* | | | *OR (95% CI)* | |  |
| **Pre-lockdown** | 0.42 | (0.30, 0.58) | *** | 0.40 | (0.28, 0.57) | *** |
| **Post-lockdown** | 0.81 | (0.72, 0.91) | *** | 0.74 | (0.68, 0.81) | *** |
| **Local authorities classified as the…** |  |  |  |  |  |  |
| …least deprived 25% (Quartile 4) | 0.99 | (0.82, 1.2) |  | 1.12 | (0.95, 1.31) |  |
| …Quartile 2 | 1.08 | (0.92, 1.27) |  | 1.18 | (1.01, 1.39) | * |
| …most deprived 25% (Quartile 1) | 1.02 | (0.81, 1.27) |  | 1.15 | (0.91, 1.46) |  |
| **Local authorities in the…** |  |  |  |  |  |  |
| …North East | 0.91 | (0.75, 1.11) |  | 0.98 | (0.75, 1.28) |  |
| …Yorkshire and the Humber | 0.60 | (0.42, 0.84) | ** | 0.68 | (0.50, 0.93) | * |
| …North West | 0.62 | (0.51, 0.77) | *** | 0.70 | (0.60, 0.82) | *** |
| …East Midlands | 0.65 | (0.56, 0.75) | *** | 0.71 | (0.62, 0.81) | *** |
| …West Midlands | 0.79 | (0.7, 0.9) | *** | 0.86 | (0.78, 0.95) | ** |
| …South West | 0.82 | (0.73, 0.92) | *** | 0.78 | (0.71, 0.85) | *** |
| …South East | 0.91 | (0.66, 1.27) |  | 0.79 | (0.59, 1.06) |  |
| …East of England | 0.88 | (0.64, 1.22) |  | 0.74 | (0.55, 1.00) |  |
| **Quartile 4 local authorities in the…** |  |  |  |  |  |  |
| …North East | NA^2^ |  |  | NA^2^ |  |  |
| …Yorkshire and the Humber | 2.00 | (1.28, 3.11) | ** | 1.59 | (1.04, 2.42) | * |
| …North West | 1.40 | (0.86, 2.28) |  | 1.44 | (0.98, 2.10) |  |
| …East Midlands | 1.05 | (0.83, 1.32) |  | 0.94 | (0.77, 1.15) |  |
| …West Midlands | 1.16 | (0.94, 1.43) |  | 1.05 | (0.87, 1.26) |  |
| …South West | 1.29 | (1.03, 1.61) | * | 1.37 | (1.12, 1.67) | ** |
| …South East | 1.28 | (0.85, 1.94) |  | 1.50 | (1.00, 2.25) | * |
| …East of England | 1.08 | (0.75, 1.56) |  | 1.29 | (0.92, 1.81) |  |
| **Quartile 3 local authorities in…** |  |  |  |  |  |  |
| …North East | 1.17 | (0.73, 1.87) |  | 1.07 | (0.64, 1.78) |  |
| …Yorkshire and the Humber | 1.02 | (0.71, 1.46) |  | 0.86 | (0.61, 1.22) |  |
| …North West | 1.72 | (1.33, 2.22) | *** | 1.86 | (1.41, 2.45) | *** |
| …East Midlands | 1.19 | (0.92, 1.53) |  | 1.15 | (0.89, 1.49) |  |
| …West Midlands | 1.49 | (1.22, 1.84) | *** | 1.40 | (1.13, 1.73) | ** |
| …South West | 1.14 | (0.87, 1.51) |  | 1.11 | (0.89, 1.38) |  |
| …South East | 1.02 | (0.69, 1.5) |  | 1.03 | (0.73, 1.46) |  |
| …East of England | 1.00 | (0.67, 1.5) |  | 1.21 | (0.82, 1.78) |  |
| **Quartile 2 local authorities in…** |  |  |  |  |  |  |
| …North East | 0.85 | (0.64, 1.15) |  | 0.72 | (0.51, 1.03) |  |
| …Yorkshire and the Humber | 1.20 | (0.8, 1.82) |  | 1.04 | (0.68, 1.59) |  |
| …North West | 1.17 | (0.81, 1.69) |  | 1.17 | (0.82, 1.65) |  |
| …East Midlands | 1.50 | (1.18, 1.91) | ** | 1.41 | (1.09, 1.83) | ** |
| …West Midlands | 1.09 | (0.82, 1.43) |  | 1.04 | (0.75, 1.44) |  |
| …South West | 1.08 | (0.86, 1.36) |  | 1.09 | (0.83, 1.44) |  |
| …South East | NA^2^ |  |  | NA^2^ |  |  |
| …East of England | NA^2^ |  |  | NA^2^ |  |  |
| * *p* ≤ 0.05, ** *p* ≤ 0.01, *** *p* ≤ 0.001 | | | | | | |
| NA^2^ = data not available; no local authorities in region classified as deprivation quartile. | | | | | | |

S6 Exchanging region reference category

|  | | | | | | |
| --- | --- | --- | --- | --- | --- | --- |
|  | **Pre-school Booster** | | | **MMR** | | |
| (ref. North West, Quartile 3) | *OR (95% CI)* | | | *OR (95% CI)* | |  |
| **Pre-lockdown** | 1.02 | (0.65, 1.6) |  | 1.15 | (0.80, 1.65) |  |
| **Post-lockdown** | 0.94 | 0.85, 1.05) |  | 1.14 | (0.95, 1.37) |  |
| **Local authorities classified as the…** |  |  |  |  |  |  |
| …least deprived 25% (Quartile 4) | 0.75 | (0.49, 1.15) |  | 0.73 | [0.50, 1.05] |  |
| …Quartile 2 | 0.64 | (0.5, 0.83) | *** | 0.61 | [0.46, 0.82] | *** |
| …most deprived 25% (Quartile 1) | 0.54 | (0.44, 0.65) | *** | 0.45 | [0.36, 0.57] | *** |
| **Local authorities in the…** |  |  |  |  |  |  |
| …North East | 0.99 | (0.65, 1.52) |  | 0.8 | (0.51, 1.26) |  |
| …Yorkshire and the Humber | 0.57 | (0.51, 0.63) | *** | 0.45 | (0.38, 0.54) | *** |
| …East Midlands | 0.71 | (0.58, 0.88) | ** | 0.62 | (0.48, 0.8) | *** |
| …West Midlands | 1.11 | (0.94, 1.3) |  | 0.93 | (0.74, 1.16) |  |
| …South West | 0.87 | (0.68, 1.12) |  | 0.66 | (0.52, 0.84) | *** |
| …South East | 0.87 | (0.71, 1.06) |  | 0.63 | (0.5, 0.79) | *** |
| …East of England | 0.82 | (0.65, 1.04) |  | 0.69 | (0.52, 0.91) | ** |
| …London | 0.93 | (0.8, 1.09) |  | 0.77 | (0.61, 0.96) | * |
| **Quartile 4 local authorities in the…** |  |  |  |  |  |  |
| …North East | NA^2^ |  |  | NA^2^ |  |  |
| …Yorkshire and the Humber | 2.40 | (1.47, 3.93) | *** | 2.38 | (1.53, 3.71) | *** |
| …East Midlands | 1.08 | (0.68, 1.73) |  | 1.06 | (0.70, 1.61) |  |
| …West Midlands | 0.95 | (0.6, 1.49) |  | 0.97 | (0.65, 1.45) |  |
| …South West | 1.38 | (0.84, 2.27) |  | 1.59 | (1.05, 2.42) | * |
| …South East | 1.54 | (0.93, 2.56) |  | 1.88 | (1.18, 2.99) | ** |
| …East of England | 1.32 | (0.81, 2.13) |  | 1.38 | (0.90, 2.14) |  |
| …London | 1.22 | (0.77, 1.95) |  | 1.29 | (0.85, 1.97) |  |
| **Quartile 3 local authorities in…** |  |  |  |  |  |  |
| …North East | 1.07 | (0.65, 1.76) |  | 1.08 | (0.65, 1.82) |  |
| …Yorkshire and the Humber | 1.73 | (1.3, 2.31) | *** | 1.92 | (1.37, 2.70) | *** |
| …East Midlands | 1.86 | (1.36, 2.53) | *** | 1.96 | (1.40, 2.76) | *** |
| …West Midlands | 1.06 | (0.77, 1.47) |  | 1.18 | (0.80, 1.75) |  |
| …South West | 1.39 | (0.99, 1.96) |  | 1.57 | (1.10, 2.24) | * |
| …South East | 1.44 | (0.96, 2.16) |  | 1.54 | (1.06, 2.24) | * |
| …East of England | 1.46 | (0.96, 2.22) |  | 1.32 | (0.88, 2.00) |  |
| …London | 1.47 | (1.05, 2.05) | * | 1.6 | (1.08, 2.35) | * |
| **Quartile 2 local authorities in…** | 1.47 | (0.91, 2.39) |  |  |  |  |
| …North East | 1.69 | (1.16, 2.47) | ** | 1.74 | (1.02, 2.98) | * |
| …Yorkshire and the Humber | 1.45 | (1.09, 1.93) | ** | 2.15 | (1.48, 3.13) | *** |
| …East Midlands | 1.15 | (0.91, 1.46) |  | 1.62 | (1.19, 2.19) | ** |
| …West Midlands | 1.51 | (1.11, 2.04) | ** | 1.33 | (1.02, 1.73) | * |
| …South West | 1.08 | (0.68, 1.73) |  | 1.68 | (1.28, 2.20) | *** |
| …South East | NA^2^ |  |  | NA^2^ |  |  |
| …East of England | NA^2^ |  |  | NA^2^ |  |  |
| …London | 1.72 | (1.33, 2.22) | *** | 1.86 | (1.41, 2.45) | *** |
| * *p* ≤ 0.05, ** *p* ≤ 0.01, *** *p* ≤ 0.001 | | | | | | |
| NA^2^ = data not available; no local authorities in region classified as deprivation quartile. | | | | | | |

S7: Linear piecewise terms

|  | | | | | | |
| --- | --- | --- | --- | --- | --- | --- |
|  | **Pre-school Booster** | | | **MMR** | | |
| (ref. London, Quartile 3) | *OR (95% CI)* | | | *OR (95% CI)* | |  |
| **Pre-lockdown** | 0.66 | (0.34, 1.3) |  | 0.63 | (0.31, 1.28) |  |
| **Post-lockdown** | 0.88 | (0.78, 0.98) | * | 0.87 | (0.76, 1.00) | * |
| **Local authorities classified as the…** |  |  |  |  |  |  |
| …least deprived 25% (Quartile 4) | 0.93 | (0.77, 1.12) |  | 0.95 | (0.79, 1.15) |  |
| …Quartile 2 | 0.94 | (0.75, 1.18) |  | 0.98 | (0.75, 1.27) |  |
| …most deprived 25% (Quartile 1) | 0.92 | (0.78, 1.08) |  | 0.84 | (0.71, 0.99) | * |
| **Local authorities in the…** |  |  |  |  |  |  |
| …North East | 1.07 | (0.70, 1.65) |  | 1.06 | (0.68, 1.65) |  |
| …Yorkshire and the Humber | 0.61 | (0.54, 0.68) | *** | 0.59 | (0.51, 0.68) | *** |
| …North West | 1.08 | (0.93, 1.25) |  | 1.31 | (1.05, 1.65) | * |
| …East Midlands | 0.77 | (0.62, 0.95) | * | 0.82 | (0.65, 1.02) |  |
| …West Midlands | 1.18 | (1.01, 1.39) | * | 1.21 | (1.00, 1.45) | * |
| …South West | 0.95 | (0.73, 1.23) |  | 0.87 | (0.71, 1.07) |  |
| …South East | 0.95 | (0.77, 1.16) |  | 0.83 | (0.68, 1.00) | * |
| …East of England | 0.89 | (0.71, 1.13) |  | 0.91 | (0.71, 1.16) |  |
| **Quartile 4 local authorities in the…** |  |  |  |  |  |  |
| …North East | NA^2^ |  |  | NA^2^ |  |  |
| …Yorkshire and the Humber | 1.97 | (1.45, 2.68) | *** | 1.85 | (1.35, 2.54) | *** |
| …North West | 0.79 | (0.50, 1.26) |  | 0.75 | (0.49, 1.15) |  |
| …East Midlands | 0.88 | (0.67, 1.16) |  | 0.82 | (0.63, 1.07) |  |
| …West Midlands | 0.77 | (0.61, 0.97) | * | 0.74 | (0.58, 0.94) | * |
| …South West | 1.12 | (0.81, 1.55) |  | 1.23 | (0.94, 1.61) |  |
| …South East | 1.25 | (0.90, 1.73) |  | 1.45 | (1.04, 2.02) | * |
| …East of England | 1.06 | (0.79, 1.42) |  | 1.06 | (0.79, 1.42) |  |
| **Quartile 3 local authorities in…** |  |  |  |  |  |  |
| …North East | 0.73 | (0.44, 1.18) |  | 0.67 | (0.40, 1.10) |  |
| …Yorkshire and the Humber | 1.18 | (0.91, 1.54) |  | 1.21 | (0.88, 1.65) |  |
| …North West | 0.67 | (0.48, 0.95) | * | 0.61 | (0.42, 0.90) | * |
| …East Midlands | 1.25 | (0.94, 1.68) |  | 1.21 | (0.88, 1.66) |  |
| …West Midlands | 0.72 | (0.54, 0.96) | * | 0.73 | (0.51, 1.04) |  |
| …South West | 0.93 | (0.66, 1.29) |  | 0.97 | (0.70, 1.36) |  |
| …South East | 0.98 | (0.66, 1.46) |  | 0.96 | (0.68, 1.37) |  |
| …East of England | 0.98 | (0.65, 1.48) |  | 0.82 | (0.55, 1.22) |  |
| **Quartile 2 local authorities in…** |  |  |  |  |  |  |
| …North East | 0.86 | (0.54, 1.38) |  | 0.93 | (0.55, 1.56) |  |
| …Yorkshire and the Humber | 0.99 | (0.69, 1.43) |  | 1.17 | (0.82, 1.65) |  |
| …North West | 0.58 | (0.45, 0.75) | *** | 0.53 | (0.40, 0.70) | *** |
| …East Midlands | 0.85 | (0.65, 1.10) |  | 0.87 | (0.66, 1.13) |  |
| …West Midlands | 0.68 | (0.55, 0.83) | *** | 0.72 | (0.58, 0.89) | ** |
| …South West | 0.89 | (0.67, 1.19) |  | 0.90 | (0.72, 1.13) |  |
| …South East | NA^2^ |  |  | NA^2^ |  |  |
| …East of England | NA^2^ |  |  | NA^2^ |  |  |
| * *p* ≤ 0.05, ** *p* ≤ 0.01, *** *p* ≤ 0.001 | | | | | | |
| NA^2^ = data not available; no local authorities in region classified as deprivation quartile. | | | | | | |

S8: Pre-school booster vaccination coverage from July – September 2014 to October – December 2022 across regions and deprivation quartiles (September 2019 is missing data).


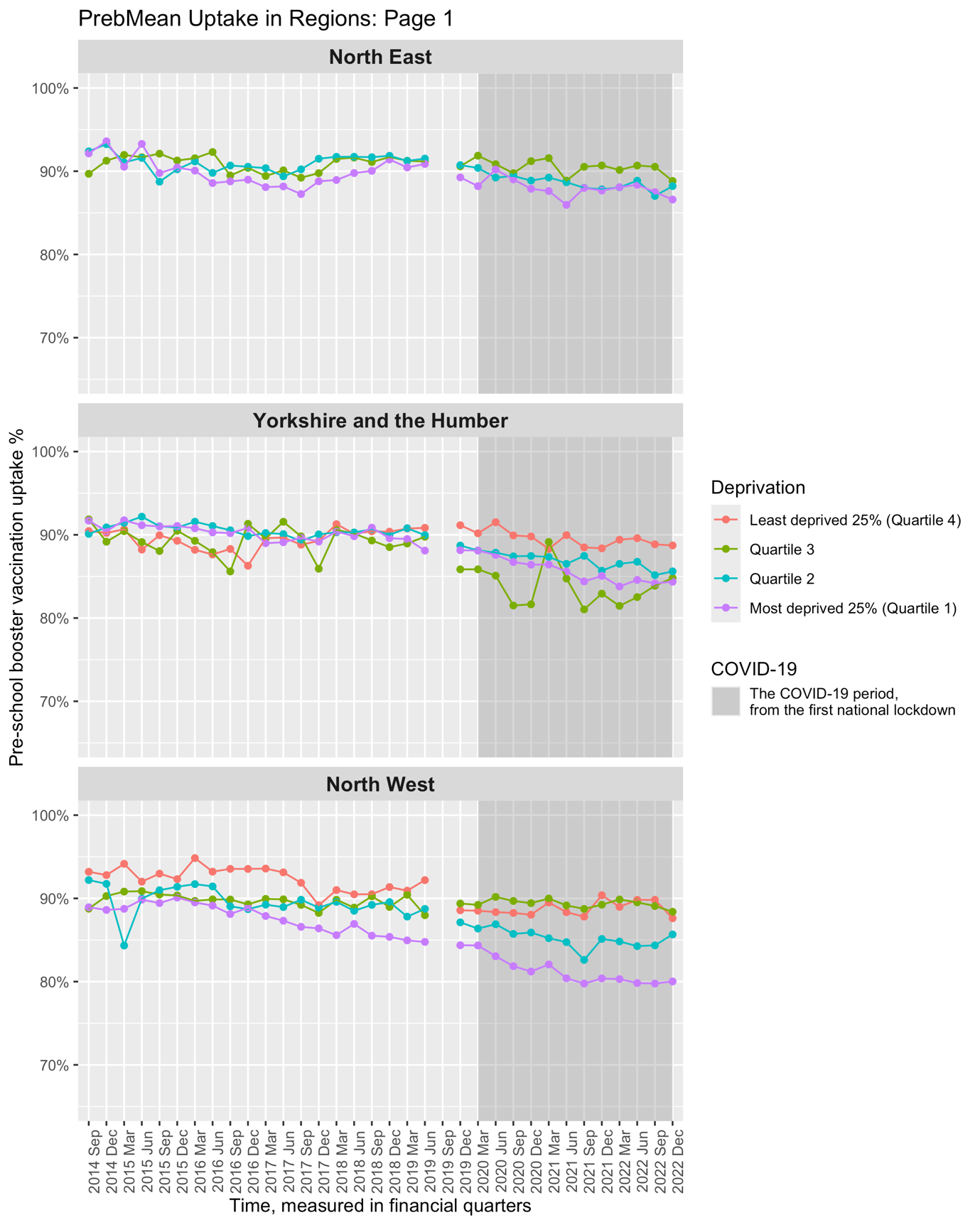


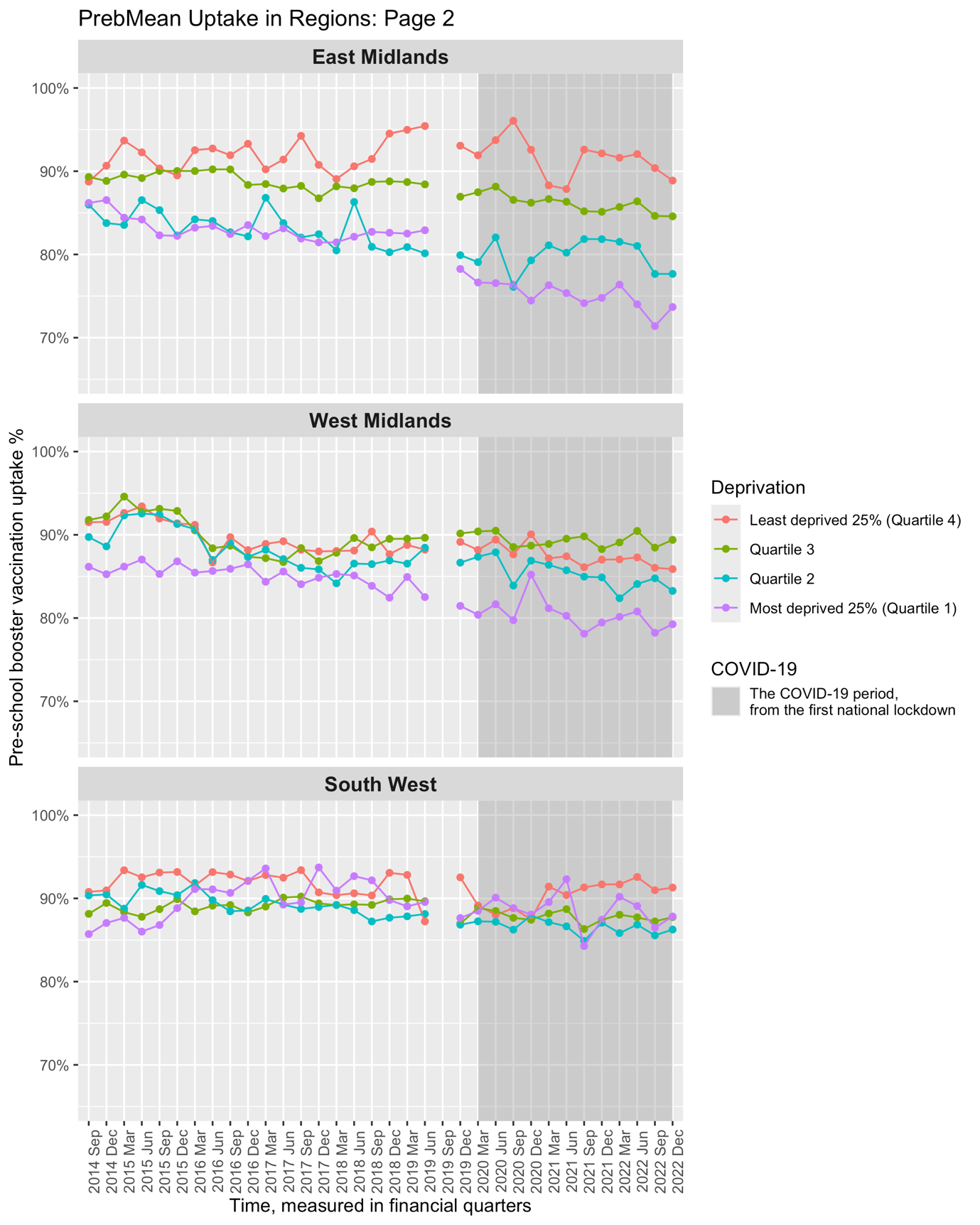


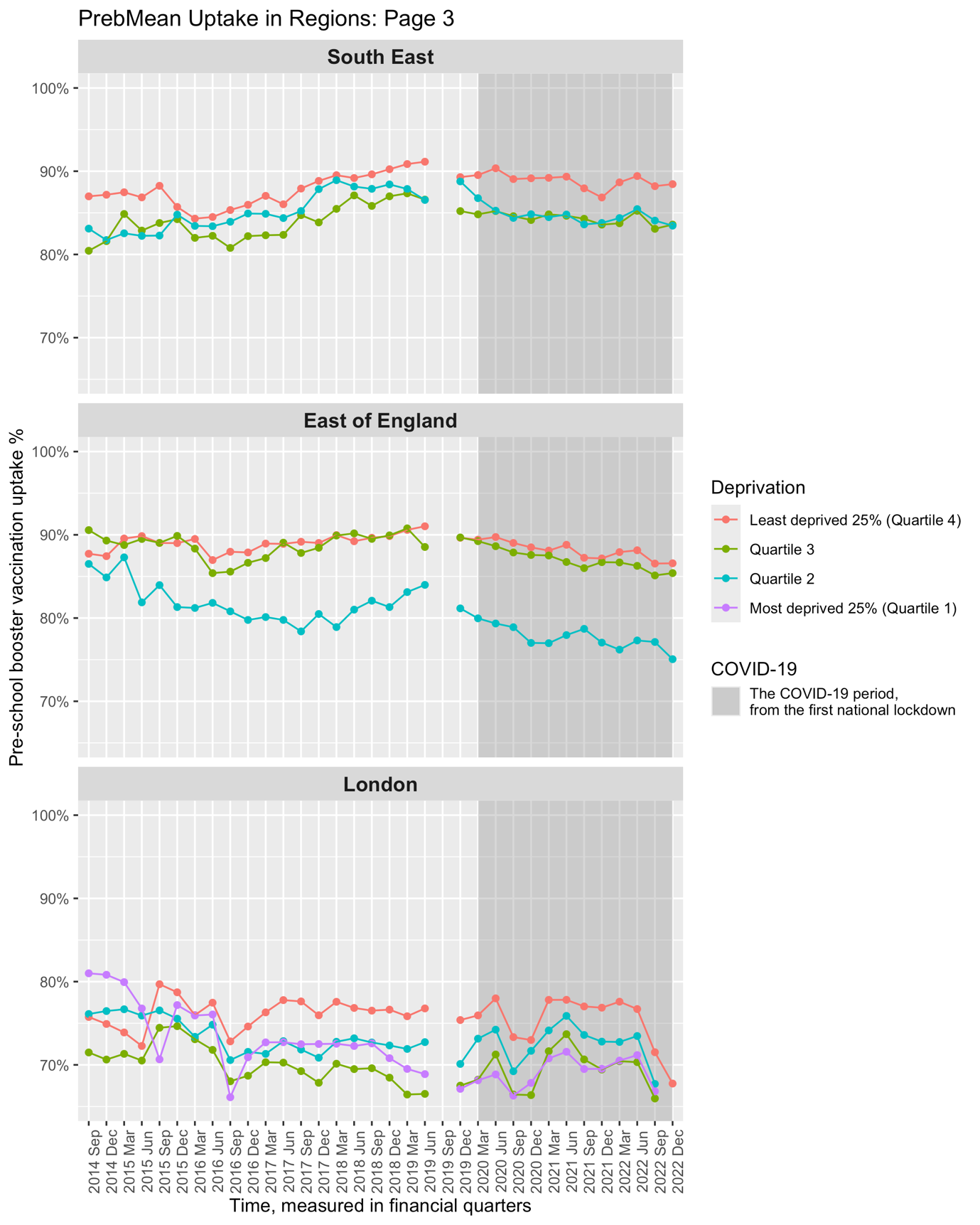


S9: MMR (measles, mumps, and rubella) vaccination coverage from July – September 2014 to October – December 2022 across regions and deprivation quartiles (September 2019 is missing data).


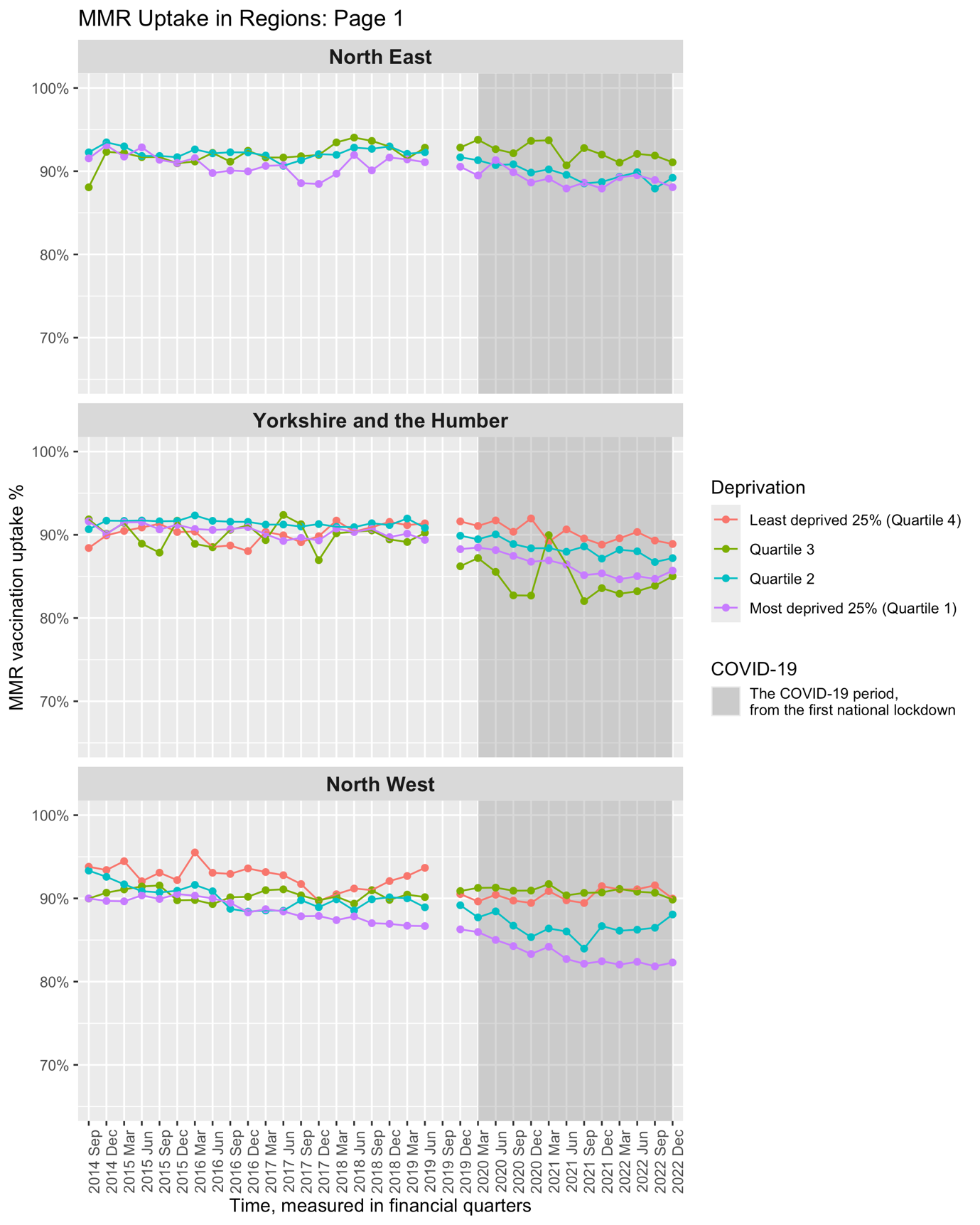


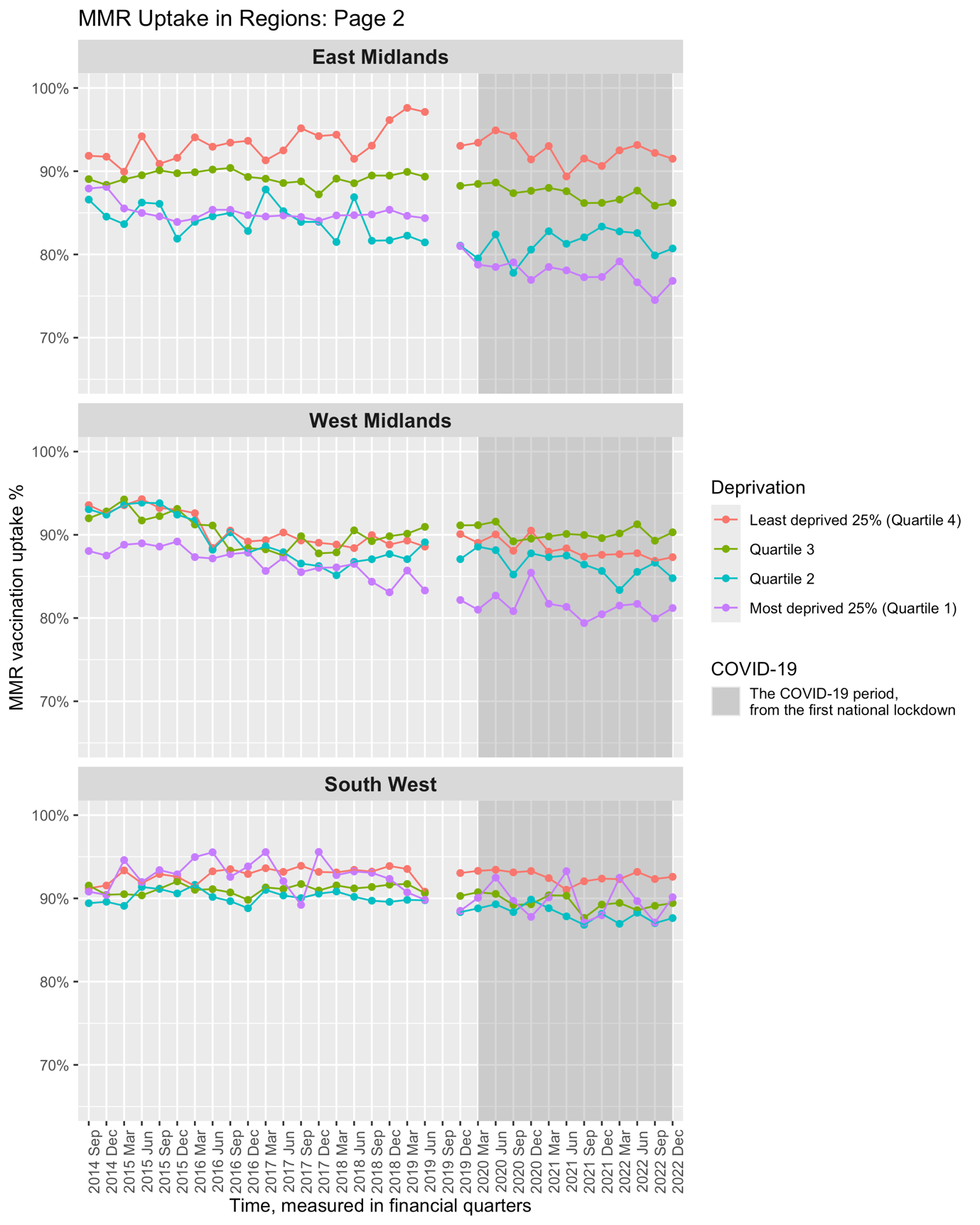


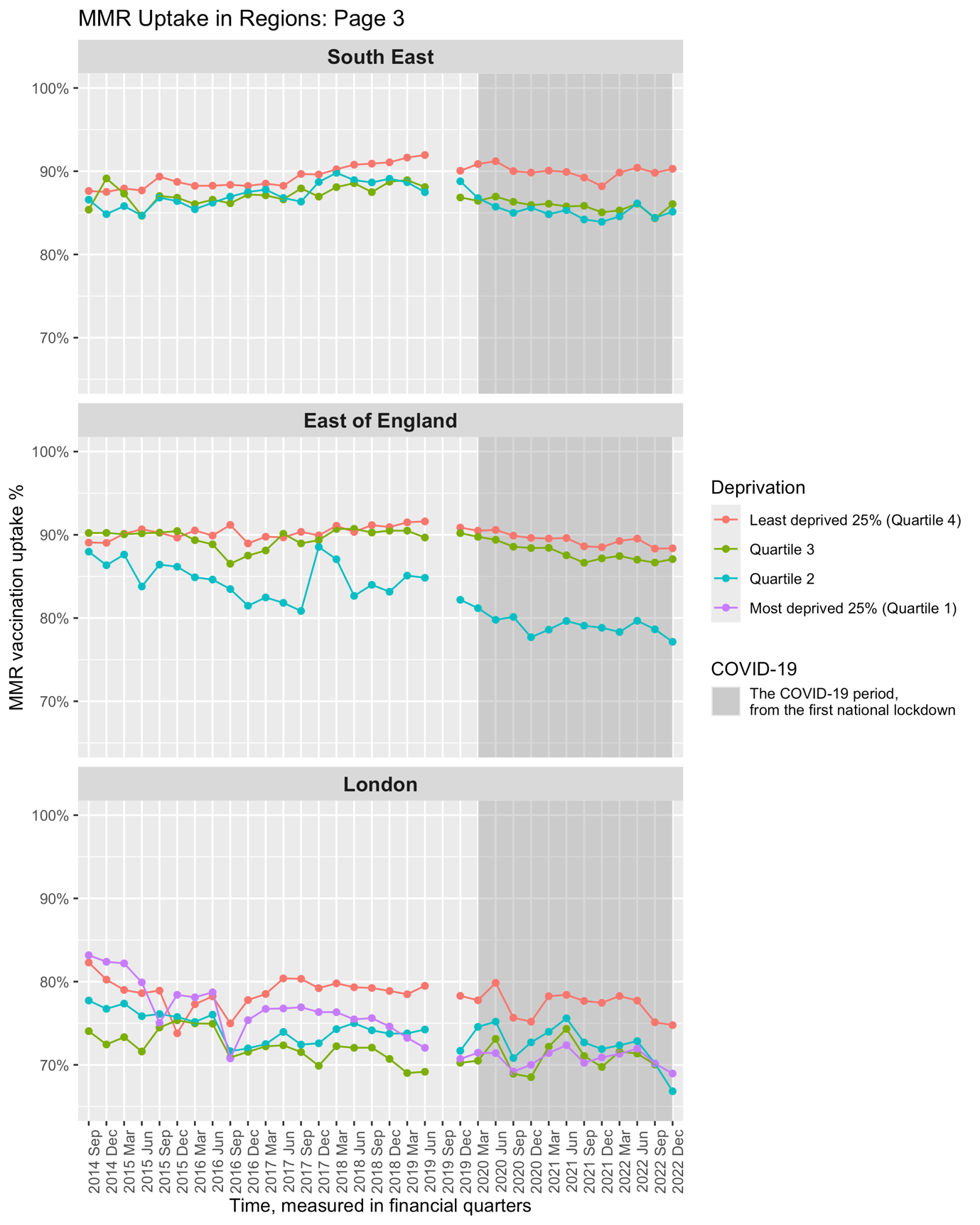

Supplement: Supplementary file 1 — Supplementary Material 1. [file 12889_2025_24207_MOESM1_ESM.docx]
